# Supplementary material for: Mass media campaigns and the ‘file drawer problem’: A mixed methods study of how to avoid campaign failure
Source: PLoS One. 2024 Apr 16;19(4):e0294372. doi: 10.1371/journal.pone.0294372 (PMC11020842; doi:10.1371/journal.pone.0294372)

# The Social Marketing ‘file drawer problem’: summary of results from Rounds 1 and 2

## Introduction to this document

This document summarises results from rounds 1 and 2 of this Delphi study. The summary is intended to inform round 3 of the study; as such, the results should be considered provisional. Data from the Round 1 interviews were analysed thematically, while Round 2 survey data were analysed descriptively. Note that the small sample size precluded any statistical analysis.

## Description of Round 2 participants

We received 28 responses to the survey (from 30 invitations). Ten participants characterised their role as being a researcher or academic (i.e. those with responsibility for research and/or evaluation of campaigns), 10 as a pracademic (i.e. those with responsibility for both campaign design and delivery and research and evaluation of campaigns), 7 as practitioner (i.e. those with responsibility for campaign design and delivery), and 1 as ‘other’. Most (19) had more than 15 years’ experience in social marketing and mass media campaigns, while the remainder had between 5- and 15-years’ experience.

## Categories of failure

Based on the Round 1 interviews, we concluded that there were three overarching categories of failure: strategic failures (relating to the decision to conduct a campaign and how it may relate to other initiatives), process failures (relating to campaign governance and how the campaign is managed from conception through evaluation), and implementation failures (relating to the actual creative design and implementation of the campaign). It also appears that implementation failures are the consequences of or are driven by strategic and process failures.

Participants were asked which of these categories of failure they felt was most significant with regards to its impact on campaigns: 12 selected strategic failures, 7 selected process failures, and 7 selected implementation failures (with 2 participants not responding). Although statistical analysis was not possible, most researchers selected strategic failures, while most practitioners selected process or implementation failures (pracademics had no identifiable pattern of response). There were no obvious differences by level of experience.

Each category of failure had several subcategories (see Figure S1). Participants were asked to rank each of these subcategories with regards to their significance (how much of an impact this failure could have on a campaign’s implementation, recognition, and/or effectiveness) and frequency (how common this failure is in campaigns). So that the rankings were on a comparable scale, we converted them into scores out of 100. Participants were also asked how amenable to change each failure was and to identify the top 3 drivers for the implementation failures. The results from these questions are summarised in the figures on the following pages.

Figure S1 Failure subcategories, their perceived significance of impact on campaigns, and their relationship with one another

*
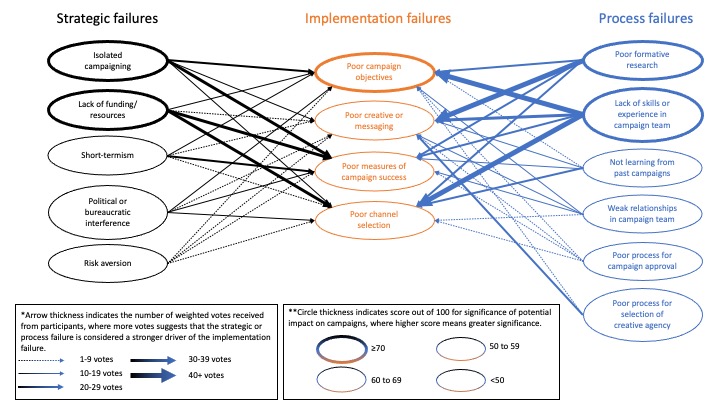
*

Figure S2 Number of times each strategic (black), process (blue), and implementation (orange) failure subcategories were ranked as the most significant (in terms of their potential impact on a campaign) and most frequent (how often they occur in campaigns) failure type


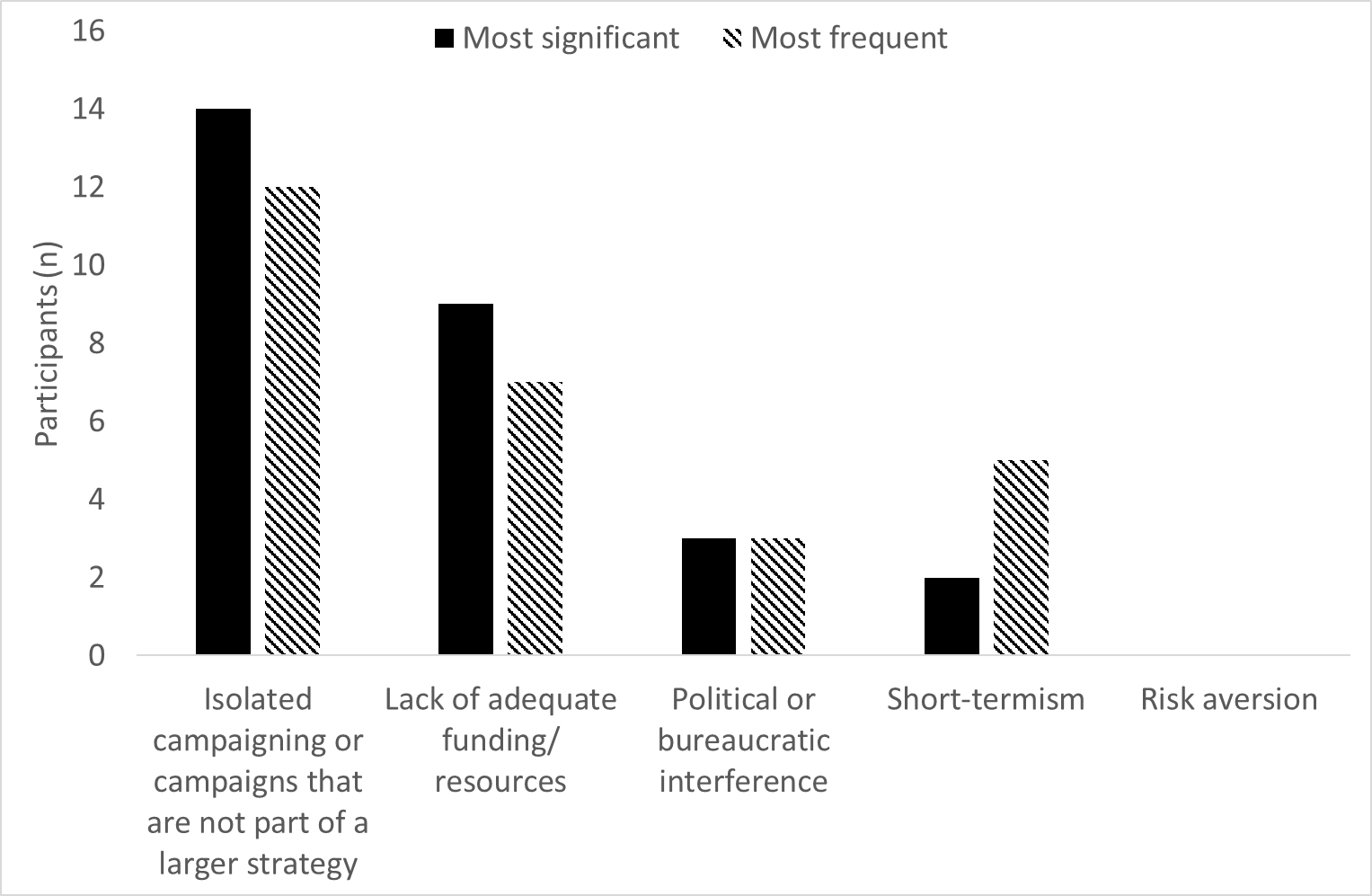

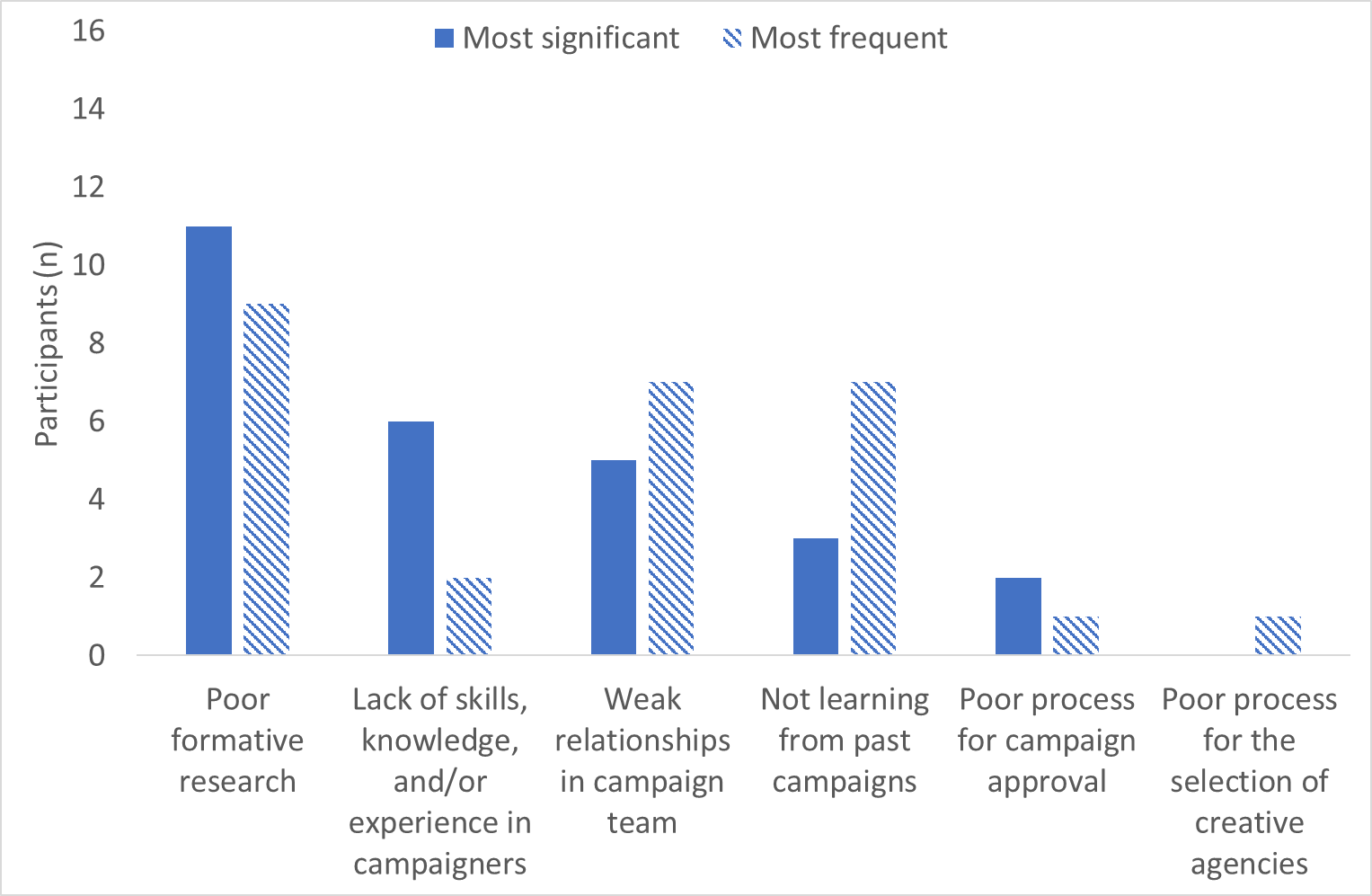


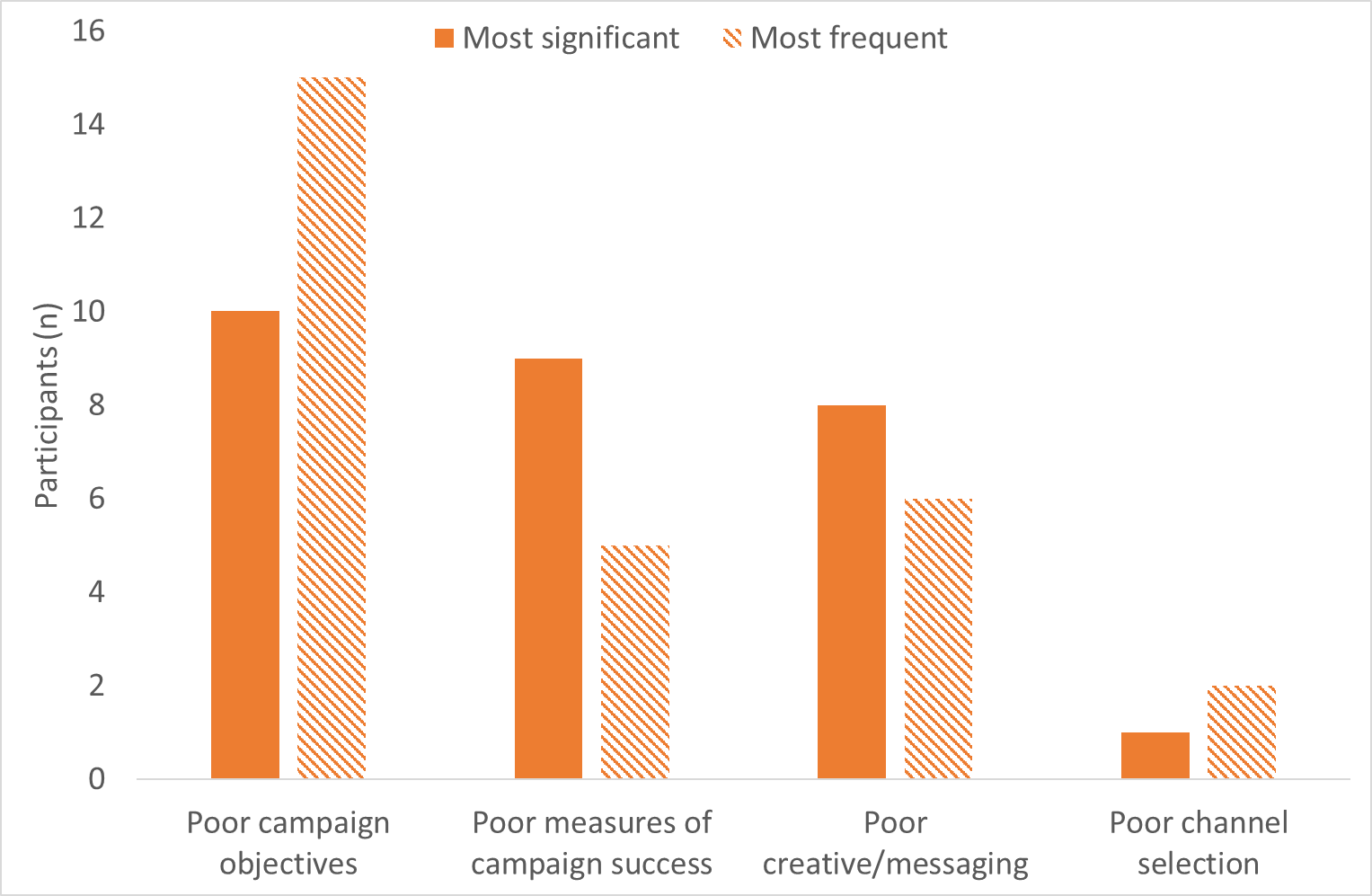


Figure S3 Quadrant map of perceived significance of impact and frequency for the failure types


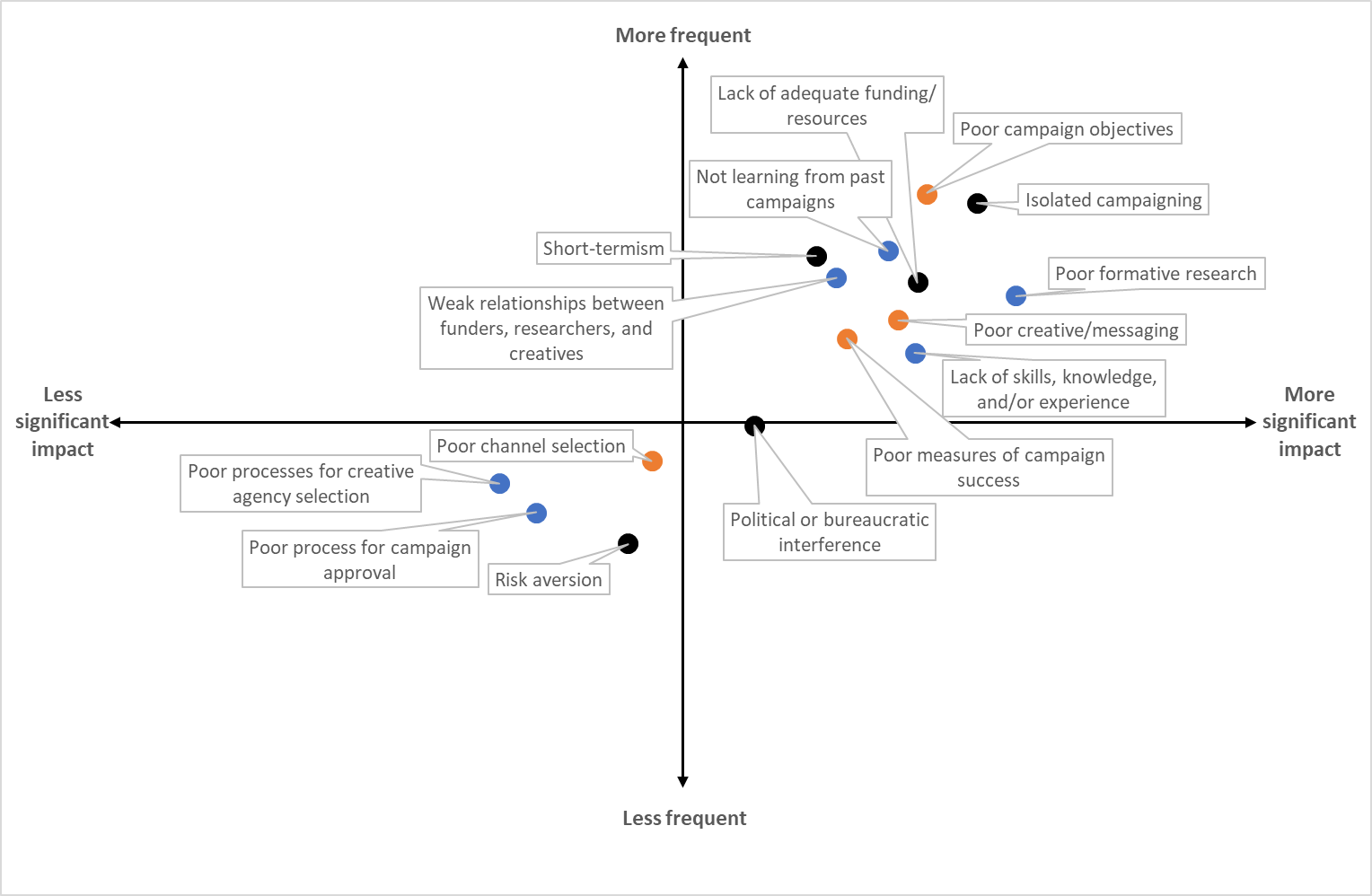


Figure S4 Quadrant map of perceived significance of impact and amenity to change for the failure types


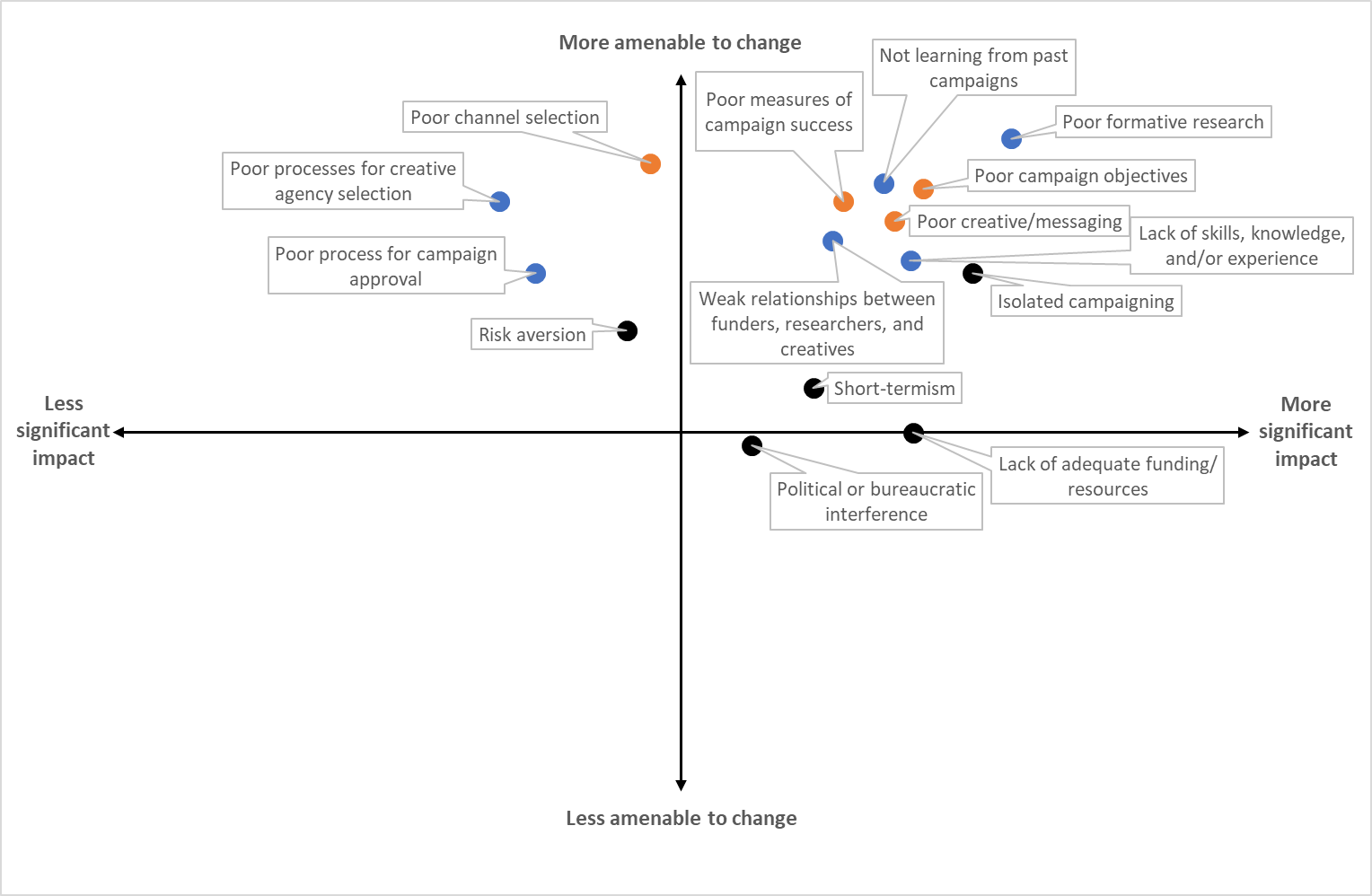

Supplement: S1 File — (DOCX) [file pone.0294372.s001.docx]
