# Supplementary material for: Mass media campaigns and the ‘file drawer problem’: A mixed methods study of how to avoid campaign failure
Source: PLoS One. 2024 Apr 16;19(4):e0294372. doi: 10.1371/journal.pone.0294372 (PMC11020842; doi:10.1371/journal.pone.0294372)
Supplement: S2 File — (DOCX) [file pone.0294372.s002.docx]

# Round one interview discussion guide

## Warm-up

This interview is about mass media-based social marketing campaigns in non-communicable disease prevention. These are initiatives that include a communications campaign, disseminated via mass media such as television, billboards, and social media. They usually aim to build knowledge, change attitudes, and/or change behaviours to ultimately improve health outcomes. They are sometimes complemented by other initiatives, such as environmental, policy, or community-based initiatives.

Can you tell me about your experience with such campaigns?

*Probing questions:*

- *What sort of campaigns have you been involved with? How long have you been involved with campaigns of this nature?*
- *What was your role in these campaigns?*
- *What did these campaigns aim to do? Do you think those aims were the right aims?*
- *In your opinion, did they achieve these aims? How do you think the campaign met its objectives? What else was good about the campaign?*
- *How did you know what happened? What information did you have on the outcomes of the campaign?*

## Experience with failed campaigns

Next, I’d like to focus on campaigns or aspects of campaigns that did not work as intended, failed to achieve their aims, or had adverse effects. Have you been involved in any such campaigns? Can you just walk me though what that was about?

*Probing questions, if yes:*

- *What was your role?*
- *What did the campaign(s) aim to do and what actually happened? Why do you think that happened?*
- *At what point was it clear that [component] had not worked as it should? Was there any opportunity to remedy that?*
- *How did you know the campaign didn’t work/failed? i.e. what did the evaluation tell you and why was that interpreted as a failed campaign (fully or partially failed)? What other information did you use to decide the campaign failed?*
- *What would you do differently if you could run it/them again? Do you think what happened in this case is typical of running these sorts of campaigns? Why was it the same/different in this case?*
- *Are there any “lessons learnt” from this that were incorporated into conducting later campaigns? [If yes, what and did it change anything?] [If no, why not do you think?]*

*Probing questions, if no:*

- *Why do you think you have been able to get consistently good results?*
- *Is there anything you think could be done to improve the results you have achieved?*

## Why do campaigns fail?

Thinking generally about the design, implementation, and/or evaluation of campaigns, where do you think they are most vulnerable in terms of achieving their aims?

*Probing questions:*

- *Are there common mistakes that campaign designers/implementers/evaluators make?*
- *In what context(s) are these mistakes most commonly made? Are there any circumstances which you think makes it more likely that [mistake] would happen in a campaign? Do you think that [circumstance] could be changed? In what way?*
- *What about issues of:*
  - *Development of campaign objectives?*
  - *Funding/resourcing?*
  - *Duration/intensity?*
  - *Message design?*
  - *Creative execution?*
  - *Measurement of impact, incl. evaluation design, methods used, indicators used etc.?*
  - *Social/political/cultural environment?*
- *Do you think there are any types of evaluation (testing or monitoring) which might help with these problems?*
- *What are the best methods for avoiding campaign failure?*
- *Are there techniques or strategies that could be used to lessen the vulnerabilities you referred to?*
- *If you had total control running a campaign, what would you do in an ideal world to ensure it had the best chance of success?*

## Wrap-up

That’s the end of the interview. Was there anything else you wanted to say that we haven’t covered today?

If there are any other experts you think might be interested in this research, can you please pass on the study details to them?

Thanks very much for your time.
