# Supplementary material for: Mass media campaigns and the ‘file drawer problem’: A mixed methods study of how to avoid campaign failure
Source: PLoS One. 2024 Apr 16;19(4):e0294372. doi: 10.1371/journal.pone.0294372 (PMC11020842; doi:10.1371/journal.pone.0294372)
Supplement: S3 File — (DOCX) [file pone.0294372.s003.docx]

# Social marketing ‘file drawer’ problem – Round 2 questionnaire

*Earlier this year, you were contacted by us regarding participating in a study called “The social marketing ‘file drawer problem’: a Delphi study of what goes into the drawer and why”. This questionnaire is the second round of that study. We are seeking to identify if there is expert consensus on factors that contribute to campaign failure, how they can be avoided or the risk mitigated, and how practice can be improved. Questions are based on the first round of interviews with experts in social marketing mass media campaigns, such as yourself.*

*This questionnaire should take about 15 minutes to complete. Your responses will be anonymous and will not be linked to your interview. You are free to not answer any questions that you do not wish to and can withdraw from the study at any time, without consequence. For a full description of the study, please see the participant information statement (available here or attached to your initial invitation email).*

*Remember that this research is focused on mass media-based social marketing campaigns in non-communicable disease prevention. These are initiatives that include a communications campaign, disseminated via mass media such as television, billboards, and social media. They usually aim to build knowledge, change attitudes, and/or change behaviours to ultimately improve health outcomes. They may or may not be complemented by other initiatives, such as environmental, policy, or community-based initiatives.*

1. Interviewees in Round 1 described three main categories of failure in campaigns. We have conceptualised these as: strategic failure (relating to the overarching decision to conduct a campaign and how it may relate to other initiatives), process failure (relating to campaign governance and how the campaign is managed from conception through evaluation), and implementation failure (relating to the actual creative design and implementation of the campaign). In your experience, which of these categories of failure is *most* significant with regards to its impact on campaigns? [SINGLE RESPONSE. RANDOMISE ORDER]
   1. Strategic failures
   2. Process failures
   3. Implementation failures

## Strategic failures

1. Interviewees identified five (5) types of strategic failure that occur in campaigns. These are:

| Failure type | Illustrative quotes |
| --- | --- |
| Isolated campaigning or campaigns that are not part of a larger strategy | “One of the problems that I still see a lot of is that campaigns are seen to be to be the answer or they're there to show that we're doing something. [This is] not probably the most optimal way to think about developing and delivering them as part of an integrated overall approach… The most effective [campaigns] that we've had and we're likely to have are ones that are strategic in nature and are integral to our overall public policy change, not just tacked on.”  “[Mass media advertising] is the visible part of all this business. It's the most visible part. You need it. But it must be the roof, it must be supported with the walls. The ceiling must be supported with something. If not, it can be a beautiful view, but just that.” |
| Short-termism | “Time I think is vital here, you've got to be in this for the long run. [Coca-Cola have] been at this job for 100 years trying to get this right. And they get it a little bit more right every year. They make enormous cockups at times... but they had the strategic vision (a) to realize that they'd screwed up, and (b) to get back on course. They had an idea of their destination. I sometimes doubt that public health has that.”  “A public health intervention goes on for years, for decades because the behaviour is prevalent, complex, needs multiple strategies. If we ever can get to some of the really difficult to pass policies and tax increases, you surely need a lot of concern among the public to support for that kind of change to happen and more of a target audience wanting help. So, it's a gradual movement that happens over time.” |
| Political or bureaucratic interference | “Campaigns do come in and out of favour based on individual experiences of people up the top. I'm sure that we've all been involved in campaigns that haven't made the light of day because someone hasn't liked them… people always believe that they know what campaigns can and can't do even though they may not have any experience in that way.”  “That happens often in government as well, where going through the various levels of approval processes, a campaign creative will get watered down, and so it just doesn't have the same cut through or the same impact with the audience.” |
| Lack of adequate funding or resources to execute the campaign strategy | “If you don't have the budget to get the reach, you often get the smaller result.”  “Where I've seen campaigns fall over, it's usually because the budget and the objectives are out of whack, and therefore the strategy selection was inappropriate.” |
| Risk aversion | “Ministers do want to be seen to be doing something, [but] they want to get into a space which is uncontroversial. ‘We're going to run a nice campaign over here and that means we don't have to do anything about the [alcohol industry’s] behaviour.’ This is enticing because it is a quick fix. Much easier to run a campaign saying ‘don't drink’ than it is to set up services to help people think about their drinking and to question global corporate capitalism, which encourages us to solve our problems by buying stuff and consuming it, and questioning the basic precepts of how we organize life.”  “So that's my point, [individual behaviour change campaigns are] safe. They're often quite useless things to do, but they're very safe and everyone thinks they're great.” |

Thinking generally about campaigns, please rank these failures from 1 (most significant or most frequent) to 5 (least significant or least frequent) according to their significance (how much of an impact this failure could have on a campaign’s implementation, recognition, and/or effectiveness) and frequency (how common this failure is in campaigns). [FORCED RANKING WITHIN EACH SUB-QUESTION]

| Failure type | Significance | Frequency |
| --- | --- | --- |
| Isolated campaigning or campaigns that are not part of a larger strategy |  |  |
| Short-termism |  |  |
| Political or bureaucratic interference |  |  |
| Lack of adequate funding or resources to execute the campaign strategy |  |  |
| Risk aversion |  |  |

1. How amenable to change do you think these failures are?

| Failure type | Very amenable to change | Somewhat amenable to change | Somewhat difficult to change | Very difficult to change |
| --- | --- | --- | --- | --- |
| Isolated campaigning or campaigns that are not part of a larger strategy |  |  |  |  |
| Short-termism |  |  |  |  |
| Political or bureaucratic interference |  |  |  |  |
| Lack of adequate funding or resources to execute the campaign strategy |  |  |  |  |
| Risk aversion |  |  |  |  |

1. Interviewees identified six (6) types of process failure that occur in campaigns. These are:

| Failure type | Illustrative quotes |
| --- | --- |
| No or poor formative research | “[In formative research, we make] sure we adopt the mindset that we don't know everything and actually [the target audience] change over time. The market's changing and so forth. Just to make sure that we are getting a fresh look each time and we're not making big assumptions that are outdated. That's really important.”  “[Being surprised by a reaction to a campaign] flies in the face of the whole notion of doing your homework. When you put a campaign on air, it's an expensive business, you get one opportunity to get it right, it's too late then to be ticking boxes and doing testing, and having hindsight about whether it was a good idea or not. I mean, there are campaigns that have been pulled or which haven’t worked, but I would argue that they could have been avoided if the formative work had been done properly.” |
| Weak relationships between funders, researchers, and creatives | “There's part of me that likes the idea that, [when] you get a creative agency on board, you pitch for like-mindedness and understanding of an issue rather than pitch for a creative so that they can help you along the journey, that it becomes a relationship between the policy makers and implementers [with] the creative minds [and] the planners [and] the evaluation people, so you've got them all in the room at the same time. That you plan for the formative research alongside the evaluators, alongside the creative people as well. And that that becomes a relationship over time.”  “Again, it goes back to relationship things. So why would whoever at the health department share information on a campaign with an advertising agency they're not going to use next time? It's that relationship thing. You know, the same as when a new agency comes in and they might read the tracking data from the last campaign, but if it wasn't their ad, they're not really going to understand it in the same way. So, to me, the key issue is actually about relationships, and sharing of knowledge.” |
| Not learning from past campaigns (own or others) | “The first burst [of a campaign] had been evaluated with the pre and post and then the second burst had no evaluation, and so being able to make a decision to continue to invest in something that you don't necessarily know how it's performing is really, really challenging.”  “What I see is that all publications are about, ‘Hey, we have been really successful. We've been impressively successful with our hypothesis.’ All papers try to find out the way to say that they'd been very successful. There's no learning from that.” |
| Inappropriate or poor processes for the selection of creative agencies | “Giving [creative agencies] a briefing document, a 20-minute briefing on the campaign, and then expecting them to go and work in a silo to come back with this amazing pitch idea that they wow you with is just so unrealistic…”  “I don't know that you'll always need to have a process by which creative agencies pitch for work. There might be some benefit to do that in terms of efficiency of money and also perceptions of fairness and all those things that go around probity and procurement, but I'm not sure that you necessarily need to do it in terms of effectiveness of campaign outcomes.” |
| Inappropriate or poor process for campaign approval | “If you need 14 signatures before it even gets to the [decision maker’s] office to run a campaign, you are fighting an uphill battle.”  “There's also a notion that you need to be able to be flexible enough or adaptable enough that you can respond to changes in the media landscape. If, for example, a TV show is not reaching the audience that you thought it would and that the buyers said that it would, that you need to be able to assess that on the way and change that. [But you can’t do that when] you've got no flexibility in being able to move stuff around because… the process by which you just got [campaign] approval means that that's what you had approval for. This rigidity around what you get approval for, it's inflexible in terms of both planning for and also constantly revising. [It] means that, [although] you implement the campaign in the best way that you can, whether it's the best way possible, I'm not sure if it is.” |
| Lack of skills, knowledge, and/or experience in campaigners | “In the field of public health, if you haven't got a professional marketer in the room, then you are heading for a problem with these sorts of things. Because public health people [often] think that all they have to do is get a single burst [of campaign activity] out and hey presto, everyone will be on board… all they've got to do is tell people and then people will understand that alcohol is harmful and won't drink so much. It just doesn't work like that. So, you need a professional marketer, you need someone in the room who understands that stuff.”  “I think I'm quite unusual in having so much social marketing experience. A lot of the people in my teams often have marketing experience, but not social marketing experience. It's really quite different… [In] marketing roles, you're looking at sales, you're looking at brand impact, you're looking at the engagement metrics, which are really easy to get, but tracking the attitudes and intentions and actual behaviour is a lot more complex, and so having the rigor behind it and making sure all those elements line up so the activities you're doing will likely lead to that outcome and you've got an evaluation that is genuinely testing that can be tricky. [Having] solid evaluation partners… is pretty important and probably doesn't always happen.” |

Thinking generally about campaigns, please rank these failures from 1 (most significant or most frequent) to 6 (least significant or least frequent) according to their significance (how much of an impact this failure could have on a campaign’s implementation, recognition, and/or effectiveness) and frequency (how common this failure is in campaigns). [FORCED RANKING WITHIN EACH SUB-QUESTION]

| Failure type | Significance | Frequency |
| --- | --- | --- |
| No or poor formative research |  |  |
| Weak relationships between funders, researchers, and creatives |  |  |
| Not learning from past campaigns (own or others) |  |  |
| Inappropriate or poor processes for the selection of creative agencies |  |  |
| Inappropriate or poor process for campaign approval |  |  |
| Lack of skills, knowledge, and/or experience in campaigners |  |  |

1. How amenable to change do you think these failures are?

| Failure type | Very amenable to change | Somewhat amenable to change | Somewhat difficult to change | Very difficult to change |
| --- | --- | --- | --- | --- |
| No or poor formative research |  |  |  |  |
| Weak relationships between funders, researchers, and creatives |  |  |  |  |
| Not learning from past campaigns (own or others) |  |  |  |  |
| Inappropriate or poor processes for the selection of creative agencies |  |  |  |  |
| Inappropriate or poor process for campaign approval |  |  |  |  |
| Lack of skills, knowledge, and/or experience in campaigners |  |  |  |  |

1. Interviewees described four (4) types of implementation failure. These failures are:

| Failure type | Illustrative quotes |
| --- | --- |
| Inappropriate or poor measures of campaign success | “I have certainly been in a situation before where you get the evaluation back, and then you're looking at your objectives and you're looking at the survey results, you're going, ‘Actually, we didn't really ask the right question. That doesn't really tell us that. I still don't know. We still can't answer in a single sentence has this worked because we probably didn't ask the right thing.’"  “You know what I hate? That campaign won all sorts of awards. And it was watched by millions of people, and it was spread all over the world. It was viral. Do you know what the behavioural results of it [were]? F*** all. Nothing. It [wasn’t] effective. And yet so many people hold it up there as ‘let’s do something as good as this [because it went viral].’” |
| Inappropriate or poor channel selection | “I guess what I was talking about before is your investment across different channels and monitoring what people are watching and what they're not, and then adjusting your buy because some things are rating better than others or they're rating well but they're not reaching your target segment. And so, you adjust your investment according with that analysis.”  “In Australia, I don't think we will ever see the big TV led campaigns that we used to have again. People just don't watch TV like they used to. Everyone's watching Netflix now. So, it is a challenge to try and achieve that reach in the population using that media. And it needs to be now a combination of different media and therefore the process that you go through of development it is going to be different I think because your way into using the media is different.” |
| Inappropriate or poor campaign objectives | “Behaviour change is a complex web of influences, some of which the individual controls, but many of which are not [in their control]. That's where, if there's a criticism of campaigns, it's often about, "Well it didn't change people's behaviours." But maybe it should never have been intended to change their behaviour.”  “I think objectives often can be too vague and not specific enough, and so then when you get your evaluation back, you can't actually tell if you've had an impact on it. Or the objectives are too high level. If the objective is just ‘solve obesity,’ then that is an unrealistic objective for a single campaign.” |
| Inappropriate or poor creative/messaging | This was a campaign that was an anti-smoking campaign that was aimed at young teenagers. And it was an advert for a range of spoof products... and the joke was, you could buy these products and get all the joy of smoking without cigarettes. It was a beautiful ad [but] we duly researched [it] and the problem is that for younger teenagers – the target audience in other words – they actually didn't see the spoof quite often. They saw these as real products that they could see a real benefit from… It seemed stupid to an adult but to a 14-year-old [the products] had an appeal. So, it didn't work.  So, all the images of often white, young, very attractive, thin women in crop tops and hot pants who look very toned and muscly… but actually for women who don't look like that – and that's the vast majority of women – it's not only not motivating, it's actually demotivating and makes women feel bad about themselves and is another reason that they stop getting active. |

Thinking generally about campaigns, please rank these failures from 1 (most significant or most frequent) to 4 (least significant or least frequent) according to their significance (how much of an impact this failure could have on a campaign’s implementation, recognition, and/or effectiveness) and frequency (how common this failure is in campaigns). [FORCED RANKING WITHIN EACH SUB-QUESTION]

| Failure type | Significance | Frequency |
| --- | --- | --- |
| Inappropriate or poor measures of campaign success |  |  |
| Inappropriate or poor channel selection |  |  |
| Inappropriate or poor campaign objectives |  |  |
| Inappropriate or poor creative/messaging |  |  |

1. How amenable to change do you think these failures are?

| Failure type | Very amenable to change | Somewhat amenable to change | Somewhat difficult to change | Very difficult to change |
| --- | --- | --- | --- | --- |
| Inappropriate or poor measures of campaign success |  |  |  |  |
| Inappropriate or poor channel selection |  |  |  |  |
| Inappropriate or poor campaign objectives |  |  |  |  |
| Inappropriate or poor creative/messaging |  |  |  |  |

1. For each of these implementation failures, what do you think are the top three (3) strategic or process failures that lead to them? [RANK MAXIMUM TOP 3 FOR EACH FAILURE TYPE]

| Failure type | Isolated campaigning or campaigns that are not part of a larger strategy | Short-termism | Political or bureaucratic interference | Lack of adequate funding or resources to execute the campaign strategy | Risk aversion | No or poor formative research | Weak relationships between funders, researchers, and creatives | Not learning from past campaigns (own or others) | Inappropriate or poor processes for the selection of creative agencies | Inappropriate or poor process for campaign approval | Lack of skills, knowledge, and/or experience in campaigners | None of these |
| --- | --- | --- | --- | --- | --- | --- | --- | --- | --- | --- | --- | --- |
| Inappropriate or poor measures of campaign success |  |  |  |  |  |  |  |  |  |  |  |  |
| Inappropriate or poor channel selection |  |  |  |  |  |  |  |  |  |  |  |  |
| Inappropriate or poor campaign objectives |  |  |  |  |  |  |  |  |  |  |  |  |
| Inappropriate or poor creative/messaging |  |  |  |  |  |  |  |  |  |  |  |  |

## Other failures

1. Are there any important failure types that lead to campaign failure that are missing from the lists above?
   1. Yes
   2. No
2. [IF YES IN Q9] Please briefly describe this/these failure/s.
   1. OPEN RESPONSE
3. [IF YES IN Q9] How significant is this/are these failure/s with regards to the impact this failure could have on a campaign's implementation, recognition, and/or effectiveness?
4. Very significant
5. Somewhat significant
6. Not at all significant
7. [IF YES IN Q9] How common is this/are these failure/s?
8. Very common
9. Common
10. Rare
11. Very rare
12. Do you have any other comments you would like to make about campaign failure?
    1. OPEN RESPONSE

## Questions about you

1. With regards to your work on mass media-based social marketing campaigns, would you describe your *primary* role as being:
2. A researcher/academic i.e. someone with responsibility for research and/or evaluation of campaigns
3. A practitioner i.e. someone with responsibility for campaign design and delivery
4. A ‘pracademic’ i.e. someone who does both campaign design and delivery AND research and evaluation of campaigns
5. Other (please specify)
6. How long have you been (or were you) involved in campaigns of this nature?
7. Less than 5 years
8. 5 to 15 years
9. More than 15 years

*That’s the end of the survey. Thank you for your time.*
