# Supplementary material for: Mass media campaigns and the ‘file drawer problem’: A mixed methods study of how to avoid campaign failure
Source: PLoS One. 2024 Apr 16;19(4):e0294372. doi: 10.1371/journal.pone.0294372 (PMC11020842; doi:10.1371/journal.pone.0294372)
Supplement: S4 File — (DOCX) [file pone.0294372.s004.docx]

# Round three interview discussion guide

## Warm-up

This interview is about mass media-based social marketing campaigns in non-communicable disease prevention. These are initiatives that include a communications campaign, disseminated via mass media such as television, billboards, and social media. They usually aim to build knowledge, change attitudes, and/or change behaviours to ultimately improve health outcomes. They are often complemented by other initiatives, such as environmental, policy, or community-based initiatives.

Can you tell me about your experience with such campaigns?

*Probing questions:*

- *What sort of campaigns have you been involved with? How long have you been involved with campaigns of this nature?*
- *What was your role in these campaigns?*
- *Have you been involved in any campaigns that have failed or have had elements that did not go to plan or achieve the campaign’s objectives? What went wrong? Were these issues avoidable or predictable?*

## Review Round 1 and 2 results

[Summary of Round 1 and 2 results provided in advance of interview]

You were provided with a short summary of the results from Round 1 and 2 of this research in advance of this interview. Have you had a chance to look at those results? [*If no, briefly talk the interviewee through the summary]*

What stands out to you about the results?

*Probing questions:*

- *Do you agree with our interpretation that strategic and process failures lead to implementation failures? Does that fit with your experience? In what ways?*
- *Do you think the failures we describe accurately capture the key issues when it comes to campaign failure?*
- *Is there anything important that is missing?*
- *Does anything surprise you in the results?*
- *Some participants mentioned a change of government, tight timelines, a lack of critical analysis of the environment in which the campaign will take place, and a lack of systems perspective as things that weren’t included in our initial analysis. What do you think of factors like these?*

## Addressing failures

As you’ll see in the summary, on average, participants in Round 2 felt that most of the failure types were amenable to change. Do you agree with that?

What do you think you and people like you can do to address the failure types we’ve identified and avoid or minimise the risk of campaign failure?

*Probing questions:*

- *What needs to change?*
- *How do we get that change?*
- *Is such change likely in your opinion?*
- *What about other actors (e.g. researchers, practitioners) involved in campaigns? Who are they? What can or should they do? How much can these people influence improvements in campaign planning and implementation?*
- *What about some of the other failure types we’ve identified, especially the ones considered more significant?*

You might also notice that process and implementation failures were considered more amenable to change than strategic failures. Why do you think that is?

*Probing questions:*

- *Why do you think others may be pessimistic about the likelihood of change for strategic failures?*
- *Can anything be done to make strategic failures more amenable to change?*

## Wrap-up and close

That’s all of the questions I have for you. Is there anything you want to raise that we haven’t already covered?

Thanks very much for your time.
