# Supplementary material for: Mass media campaigns and the ‘file drawer problem’: A mixed methods study of how to avoid campaign failure
Source: PLoS One. 2024 Apr 16;19(4):e0294372. doi: 10.1371/journal.pone.0294372 (PMC11020842; doi:10.1371/journal.pone.0294372)
Supplement: Attachment — Submitted filename: Response to PLOS reviewers_File drawer paper.docx [file pone.0294372.s005.docx]

Dear Dr White,

Thank you for considering our paper “Mass media campaigns and the ‘file drawer problem’: a mixed methods study of how to avoid campaign failure” for publication in *PLOS ONE*. Thank you particularly for reviewing our paper when a second reviewer could not be found. We have responded to your comments and those of reviewer 1 as outlined in the tables below (changes have been tracked in the revised manuscript) and believe that the changes have improved the paper.

Kind regards,

Dr James Kite (on behalf of all authors)

**Reviewer 1**

| Comment | Response | Page and paragraph |
| --- | --- | --- |
| *ABSTRACT*  *- Delete “surprisingly”* | We have deleted ‘surprisingly’ as suggested. | Abstract |
| *- The methods section of the abstract is very short* | We have added additional detail on the methods to the abstract | Abstract |
| *INTRODUCTION*  *- Well written overall.* | Thank you. | - |
| *METHODS*  *- Move lines 89 to 91 up under Methods (to become line 86).* | We have moved the sentence as suggested. Note that the line numbers have changed between versions. | P5, par 2 |
| *- Heading on line 88 to be rephrased “recruitment.”* | We have modified the heading as suggested. | P5 |
| *- Recruitment is unclear. Provide a breakdown as to how each “sub-population” was recruited/approached.* | All participants were recruited in the same manner, as described in the Recruitment section. We did not apply quotas or use different methods to recruit particular sub-populations of participants. | - |
| *- It is unclear what the target sample size was, and why it was chosen.* | We did not have a target sample size for this study. This reflects our purposive sampling strategy for experts, with the aim being to collect a range of views and experiences, rather than seeking a representative sample size. We have now clarified this in the Methods. | P5, last par |
| *- Description of the sample usually belongs under results. See checklists like SRQR to guide the reporting of this paper.* | We have moved the description of the sample to the results as suggested. | P8, first par |
| *- It is unclear as to how this paper is mixed methods (as opposed to purely qualitative).* | We used both interviews and a survey, as described in the Data collection section (p6). | - |
| *- Would be good to see a table summarising participant characteristics.* | Thank you for this suggestion, but all participant characteristics that we have are already described in the text. We did not collect additional characteristics (e.g. age, gender) that could be summarised in a table. We therefore have not added an additional table as we believe it would only duplicate existing information. | - |
| *- It would be good to see a figure illustrating the various rounds with the various stakeholders (the process by which data were collected).* | We believe this is what Figure 1 displays but would be happy to modify this or consider an additional figure if we have misunderstood the reviewer’s comment. | - |
| *- There is Figure 2 but not Figure 1. Revise/proofread.* | Our apologies for this error in the Figure captions. These have been removed from the end of the document and submitted as separate files now, as per the journal’s requirements. This has removed the errant captions. | - |
| *- It is unclear how analysis was done. Was there coding? What guided the choice of themes? Were there sub-themes? Which guidelines were used? What was done to enhance trustworthiness? How many people anaylsed data? Was there cross-checking to ensure accuracy?* | We have revised the Data Collection section (now titled Data collection and analysis) to describe the analysis process in more detail. | P6, par 3 and P7 par 2 |
| *RESULTS and DISCUSSION*  *- Quite lengthy yet informative. Great to see illustrative participant excerpts in the results.* | Thank you. | - |
| *FIGURE 2 (which is probably figure 1 since there is no figure 1) is pixelated and hard to read.* | Thank you for alerting us to this issue. We will work with the journal to ensure that the final version of the required figure is of sufficient quality. | - |
| *FIGURE 3 (which will become figure 2) captures the results very well.* | Thank you. | - |

**Reviewer 2 (Academic Editor)**

| Comment | Response | Page and paragraph |
| --- | --- | --- |
| *I agree that the study adopted a sequential mixed methods design, but it used such an atypical approach to a Delphi study that it is not clear whether it can still be classified as a Delphi study. Whilst definitions vary, the Delphi technique is generally regarded as a consensus methodology in which a Panel of experts individually consider their position on aspects of a topic and in subsequent Rounds, following feedback on the collective opinion of the Panel on these aspects from the previous Round, the (remaining) Panellists reconsider their previously declared position on these aspects, towards achieving (or not achieving) consensus. No explanation or justification (e.g. using methodological literature) is provided in the paper for such deviation from even accepted modifications to a ‘standard’ Delphi approach and the study seems instead to have broadly explored the topic and refined the findings, rather than specifically achieving a pre-defined consensus on aspects of the topic.* | Thank you for identifying this issue with our description of the study as a Delphi study. We now agree that we have misdescribed our study and have changed the description of the study to be a ‘sequential mixed methods design’ as suggested. We no longer use the term Delphi study. | Title  Abstract  P5, par 3  P8, par 3  P24, par 2 |
| *An explanation is needed in the main text of how the descriptive statistical analysis of quantitative data from Round 2 informed the analysis presented in the results section.* | We have added this detail to the Mixed methods integration section. | P7, last par |
| *The description in the results of how the Round 3 data was analysed to generate the 4 major themes is a point of method and not a result.* | We have moved this description to the Methods section as advised; thank you. | P8, first par |
| *The results need to be presented more succinctly. Even for a qualitative analysis, the results section is too long.* | We have shortened the Results section as suggested. | Results |
| *The discussion section would benefit from the implications of the work being better teased out (perhaps at the end).* | As advised, we have clarified the implications of our findings in the Discussion section. | Discussion |

**Additional journal requirements**

| Comment | Response |
| --- | --- |
| *1. When submitting your revision, we need you to address these additional requirements.*  *Please ensure that your manuscript meets PLOS ONE's style requirements, including those for file naming. The PLOS ONE style templates can be found at*  *https://journals.plos.org/plosone/s/file?id=wjVg/PLOSOne_formatting_sample_main_body.pdf and*  *https://journals.plos.org/plosone/s/file?id=ba62/PLOSOne_formatting_sample_title_authors_affiliations.pdf.* | We have checked the linked documents and altered the document formatting accordingly. |
| *a) If there are ethical or legal restrictions on sharing a de-identified data set, please explain them in detail (e.g., data contain potentially identifying or sensitive patient information, data are owned by a third-party organization, etc.) and who has imposed them (e.g., a Research Ethics Committee or Institutional Review Board, etc.). Please also provide contact information for a data access committee, ethics committee, or other institutional body to which data requests may be sent.* | Sharing the data set is not possible due to ethical concerns. Specifically, the raw data are identifiable as participants described their roles in detail and referenced specific campaigns that they had worked on. Participants did not consent to sharing their interview transcripts. Data access requests could be directed to The University of Sydney’s Human Research Ethics Committee, citing project number 2020/091. We have updated the data availability statement accordingly. |
| *4. Please remove your figures from within your manuscript file, leaving only the individual TIFF/EPS image files, uploaded separately. These will be automatically included in the reviewers’ PDF.* | We have removed the figures from the submitted manuscript file and uploaded them separately. |
| *5. Please include captions for your Supporting Information files at the end of your manuscript, and update any in-text citations to match accordingly. Please see our Supporting Information guidelines for more information: http://journals.plos.org/plosone/s/supporting-information.* | As directed, we have added captions for the supporting information files to the end of the manuscript. |
